# PbXND1 Results in a Xylem-Deficient Dwarf Phenotype through Interaction with PbTCP4 in Pear (*Pyrus bretschneideri* Rehd.)

**DOI:** 10.3390/ijms23158699

**Published:** 2022-08-04

**Authors:** Yuxiong Xiao, Guangya Sha, Di Wang, Rui Gao, Bingqing Qie, Liu Cong, Rui Zhai, Chengquan Yang, Zhigang Wang, Lingfei Xu

**Affiliations:** College of Horticulture, Northwest A&F University, Taicheng Road No.3, Xianyang 712100, China

**Keywords:** dwarfing, xylem development, pear, Red Zaosu, PbXND1, PbTCP4

## Abstract

Dwarfing is an important agronomic characteristic in fruit breeding. However, due to the lack of dwarf cultivars and dwarf stocks, the dwarfing mechanism is poorly understood in pears. In this research, we discovered that the dwarf hybrid seedlings of pear (*Pyrus bretschneideri* Rehd.), ‘Red Zaosu,’ exhibited a xylem-deficient dwarf phenotype. The expression level of *PbXND1*, a suppressor of xylem development, was markedly enhanced in dwarf hybrid seedlings and its overexpression in pear results in a xylem-deficient dwarf phenotype. To further dissect the mechanism of PbXND1, PbTCP4 was isolated as a PbXND1 interaction protein through the pear yeast library. Root transformation experiments showed that PbTCP4 promotes root xylem development. Dual-luciferase assays showed that PbXND1 interactions with PbTCP4 suppressed the function of PbTCP4. PbXND1 expression resulted in a small amount of PbTCP4 sequestration in the cytoplasm and thereby prevented it from activating the gene expression, as assessed by bimolecular fluorescence complementation and co-location analyses. Additionally, PbXND1 affected the DNA-binding ability of PbTCP4, as determined by utilizing an electrophoretic mobility shift assay. These results suggest that PbXND1 regulates the function of PbTCP4 principally by affecting the DNA-binding ability of PbTCP4, whereas the cytoplasmic sequestration of PbTCP4 is only a minor factor. Taken together, this study provides new theoretical support for the extreme dwarfism associated with the absence of xylem caused by PbXND1, and it has significant reference value for the breeding of dwarf varieties and dwarf rootstocks of the pear.

## 1. Introduction

Plant height is one of the most important target traits in fruit breeding. High-density planting can save labor and improve productivity and quality. Pear is widely cultivated worldwide because of its high economic value. However, the lack of dwarf varieties and dwarf rootstocks severely restricts the intensive cultivation of pears. At present, several hypotheses are considered to be important for the dwarfing of fruit trees. On the one hand, many plant hormones, such as auxin, gibberellins (Gas) and brassinolide (BR), are reported to be involved in dwarf fruit trees and rootstock-induced dwarfing scions. Based on the importance of plant hormones, many genes related to plant hormone synthesis, degradation, transport and signal transduction have proved to be involved in the dwarfing of fruit trees. The auxin transport-related gene, *PcPIN-L*, and brassinolide biosynthesis-related gene, *PcDWF1*, are involved in the dwarf-style pear [1,2]. The inhibition of the expression of the gene encoding the gibberellin (GA) biosynthesis enzyme GA20 oxidase reduced the level of bioactive GAs, resulting in a decrease in plant height in apples and bananas [3,4]. A nonsynonymous single nucleotide mutation in GID1c disrupts its interaction with DELLA1, resulting in a GA-insensitive dwarf phenotype in peach [5]. A gibberellin-related gene, *DkGA2ox1*, was found to move from the rootstock to the scion and reduce the gibberellin content in scions, thereby resulting in interstock-induced dwarfism in Sweet Persimmon (*Diospyros kaki* Thunb.) [6]. On the other hand, xylem development is considered to be an important dwarfing factor and has become an important index for evaluating the dwarfism of fruit trees and rootstocks. Meanwhile, some theories have been proposed that seek to evaluate the dwarf rootstock based on xylem development, such as the wood/bark ratio, vessel diameter and vessel density [7,8,9]. The dwarfing rootstocks exhibit a reduction in the number and diameter of xylem cells, which leads to low water transport efficiency in apples [10]. The xylem of the dwarf pear has less lignification, smaller vessels and lower vessel density than the standard pear [11]. Therefore, strengthening research on the molecular mechanisms of dwarf varieties and dwarf rootstocks, especially the molecular mechanism of xylem development, can provide effective theoretical guidance and technical support for high-density planting.

Xylem development as an important factor affecting plant height. The xylem includes several cell types, including fiber cells, xylem parenchymal cells and tracheary elements (TEs) [12,13]. The synthesis and deposition of secondary cell wall components, including cellulose, hemicellulose and lignin, are required for xylem formation [14]. In addition, programmed cell death is involved in the differentiation of xylem vessels [15]. All of these cellular events in xylem formation are directed by similar master regulators, plant-specific NAM/ATAF/CUC (NAC) domain transcription factors. For example, *VASCULAR-RELATED NAC-DOMAIN1* (*VND*) *1–7* are identified as master regulators of xylem vessel formation, and the VND6 and VND7 proteins fusing to the SRDX strong repression domain results in a xylem-deficient dwarf phenotype [16]. There are other secondary wall NAC (SWN) genes involved in xylem development, including the *NAC secondary wall thickening-promoting factors1/2/3* (*NST1/2/3*) and the *secondary wall-associated NAC domain proteins1/2/3* (*SND1/2/3*). *NST1/2/3* promote secondary wall thickening and regulate anther dehiscence. They are also specifically involved in the secondary wall thickening of interfascicular fibers [17,18,19,20]. *SND1/2/3* are also master transcriptional switches that activate the secondary wall biosynthesis and enhanced secondary wall thickening in the fibers. SND1 silencing did not lead to stems that stand as straight as wild-type stems in *Arabidopsis* and *Populus*, and its overexpression showed significant growth retardation in *Arabidopsis* [21,22]. These SWNs induced a hierarchical network of transcriptional regulation associated with MYB-type transcription factors, such as MYB46 and MYB83, which are secondary master regulators of secondary cell wall formation. MYB46 and MYB83 silencing lead to reduced growth, with shorter inflorescence stems compared with the wild type, and their overexpression exhibited obvious stunted growth in *Arabidopsis* and apple [23,24,25,26,27,28,29]. In addition, other transcription factors are involved in xylem formation. The *knat3 knat7* double mutant inhibited xylem development and exhibited a dwarf phenotype [30]. ANAC005 overexpression resulted in a similar xylem-deficient dwarf phenotype in *Arabidopsis* [31]. TCP4 directly binds to the promoter of VND7 to activate secondary cell wall biosynthesis and programmed cell death, and to accelerate vessel formation [32]. In general, xylem development is closely related to plant height, and irregular xylem severely affects plant growth and development. 

XND1, as a negative regulator of secondary wall formation and programmed cell death, is highly expressed in xylem and during TEs differentiation [33,34]. *XND1* overexpression results in extreme dwarfism, associated with the absence of xylem vessels, and XND1 knockout plants exhibit decreased plant height and tracheary element length in *Arabidopsis thaliana* [35]. XND1 is involved in the differentiation of xylem TEs through interactions with RETINOBLASTOMA-RELATED, but the exact molecular mechanism of this interaction remains unclear [36]. KNAT2/6b directly activates the expression of PagXND1a, resulting in a xylem-deficient dwarf phenotype [37]. However, PagGRF12a directly activates the expression of PagXND1a, but does not affect the plant height in the poplar [38]. This may be related to the ability of upstream genes to promote the XND1 promoter. Different promoters expressing XND1 can produce different phenotypes, and the overexpression of XND1 under the promoter of the secondary wall-specific cellulose synthase A8 (CESA8) gene and the constitutive CaMV 35S promoter results in a xylem-deficient dwarf phenotype, while growth in Arabidopsis is not affected by the promoter of the fiber-specific SND1 gene [39].

In this study, we found that PbXND1 interacted with PbTCP4 to coordinate xylem development in the pear. *PbXND1* overexpression inhibited xylem development and reduced plant height, whereas *PbTCP4* promoted xylem development in the pear. *PbTCP4* directly activated the expression of *PbVND7*. In addition, our results demonstrated that *PbXND1* acts as a transcriptional repressor of *PbTCP4* through protein interactions. PbXND1 regulates the function of PbTCP4 primarily by affecting the DNA-binding ability of PbTCP4, and the cytoplasmic sequestration of PbTCP4 was only a minor factor. The mechanism whereby PbXND1 can affect the binding of interacting proteins to downstream gene promoters is proposed for the first time. In brief, this study provides new theoretical support for the xylem-deficient dwarf phenotype caused by PbXND1.

## 2. Results

### 2.1. Expression Patterns of PbXND1

**Figure 1:**
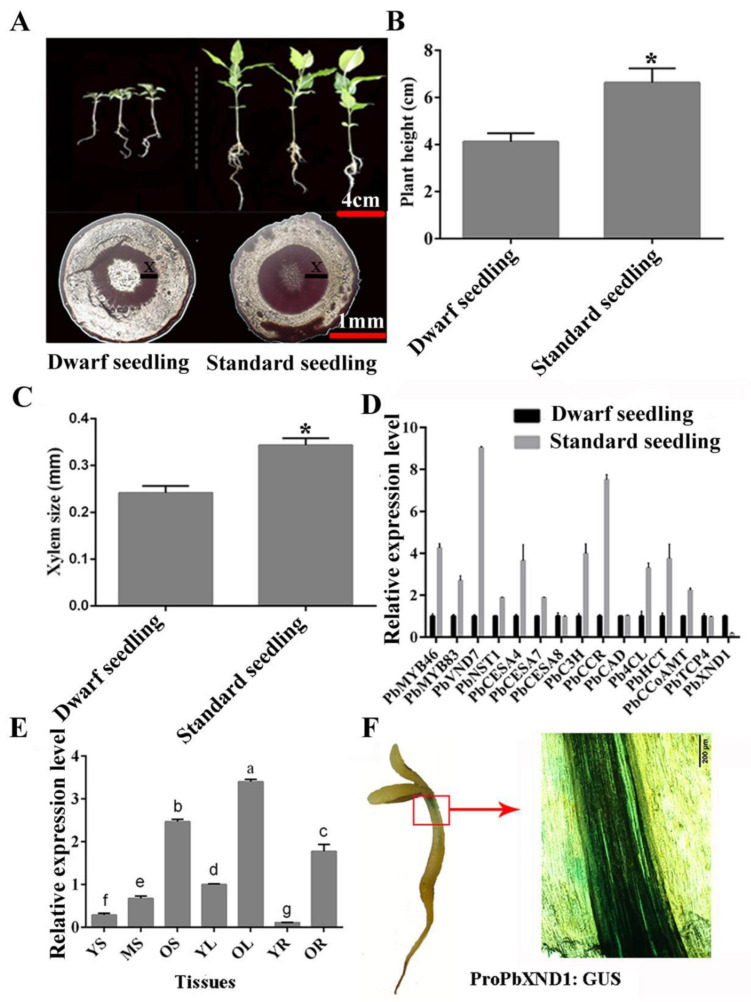
**PbXND1 isclosely correlated with xylem development.** (**A**) ‘Red Zaosu’ filial generations and phloroglucinol-HCl staining of the stem. X, xylem. Bars, 4 cm and 1 mm. (**B**) Plant height of the dwarf hybrid seedling and standard hybrid seedling. Data are shown as means ± SD (*n* = 25). Asterisks indicate significance levels. (*, *p* < 0.05). (**C**) Xylem size of the dwarf hybrid seedling and standard hybrid seedling. Data are shown as means ± SD (*n* = 3). Asterisks indicate significance levels. (*, *p* < 0.05). (**D**) Relative expression of xylem-related genes in filial generations. Data are shown as means ± SD (*n* = 3). (**E**) Expression levels of PbXND1 and PbTCP4 in different tissues and developmental stages. YL, young stem; ML, middle stem; OL, old stem; YL, young leaf; OL, old leaf; YR, young root; OR, old root. Data are shown as means ± SD (*n* = 3). Different letters denote statistical significance (*p* < 0.05). (**F**) Activity of the PbXND1 promoter in the pear vascular tissue. Bars, 200 μm.

In this study, we found that the dwarf hybrid seedlings of the ‘Red Zaosu’ pear showed obvious growth retardation and reduced plant height. Phloroglucinol-HCl staining observation and xylem measurements indicated abnormal xylem development in the dwarf hybrid seedlings (Figure 1A–C). To investigate the xylem formation-related mechanism, we analyzed the expression levels of xylem-related genes between the dwarf and the standard hybrid seedlings. The expression level of some positive regulators of xylem development was decreased, whereas the level of the negative regulator *PbXND1* was increased in the dwarf hybrid seedlings (Figure 1D). We cloned the coding sequence of *PbXND1* from the dwarf hybrid seedlings (Figure S1A). The constructed phylogenetic tree showed that PbXND1 was homologous with AtXND1, and the highly conserved NAM superfamily DNA-binding domain was identified in the PbXND1 N-terminal sequence (Figure S1B). Moreover, we analyzed the expression levels of *PbXND1* in the roots, stems and leaves of the pear at different developmental stages. The expression level of *PbXND1* was the highest in the leaves, followed by the stems, and it was the lowest in the roots. The *PbXND1* expression level increased along with plant tissue maturation and lignification (Figure 1E). Furthermore, PbXND1 was mainly expressed in the vascular tissue, as shown by a promoter GUS assay (Figure 1F). These results indicated that PbXND1 may be involved in the xylem-deficient dwarf phenotype of the dwarf hybrid seedlings.

### 2.2. PbXND1 Results in a Xylem-Deficient Dwarf Phenotype in Tobacco and Pear

**Figure 2:**
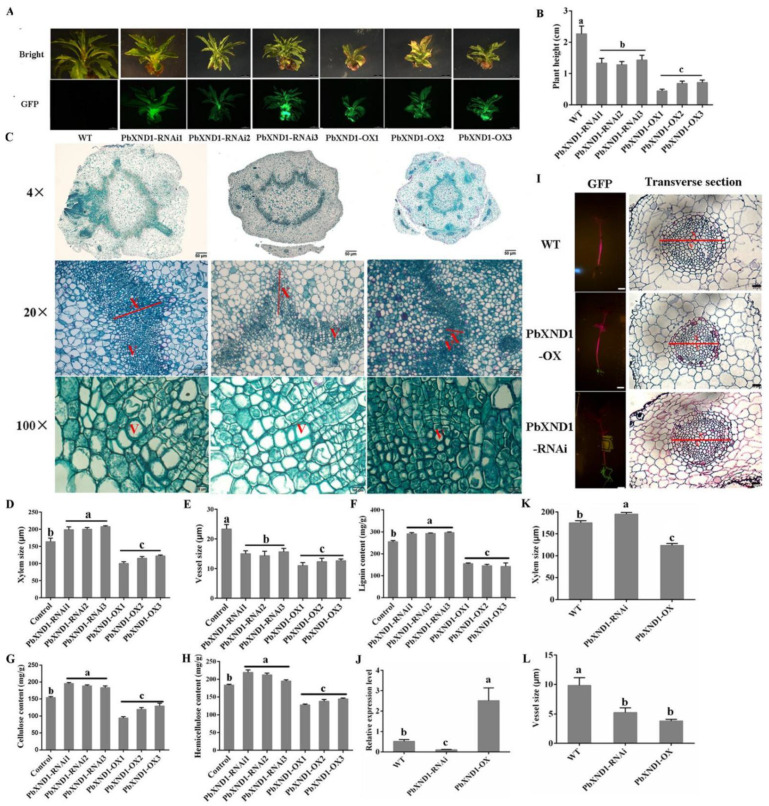
**PbXND1 inhibited xylem development in the transgenic pear.** (**A**) The growth phenotypes and green fluorescence detection of one-month-old PbXND1 overexpression and silencing pear plants and control plants. Bars, 2 mm. (**B**) Plant height of one-month-old PbXND1 overexpression and silencing pear plants and control plants. (**C**) Cross sections of the stems of pear plants stained with safranin O and fast green. X, xylem; V, vessel. Bars, 200 µm and 50 µm. Data are shown as means ± SD (*n* = 3). (**D**) Xylem size of PbXND1 overexpression and silencing pear plants. Data are shown as means ± SD (*n* = 3). (**E**) Vessel size of PbXND1 overexpression and silencing pear plants. Data are shown as means ± SD (*n* = 3). (**F**–**H**) Lignin, cellulose and hemicellulose content of PbXND1 overexpression and silencing pear plants. One month-old pear stems as experimental materials. Data are shown as means ± SD (*n* = 5). (**I**) GFP and transverse section observation of wild-type (WT), PbXND1-OX and PbTCP4-OX roots. X, xylem; V, vessel. Bars, 20 µm. (**J**) Relative expression of PbXND1 in transgenic pear roots. Data are shown as means ± SD (*n* = 3). Different letters denote statistical significance (*p* < 0.05). (**K**) Xylem size of pear roots. (**L**) Vessel size of pear roots. Data are shown as means ± SD (*n* = 3). Different letters denote statistical significance (*p* < 0.05).

Next, we verified the function of PbXND1 using transgenic technology. The PbXND1 transgenic lines of tobacco and pear were obtained after characterization using GFP fluorescence and quantitative analyses (Figure S2, Figure 2A and Figure S3A,B). To understand the mechanism by which PbXND1 affects stem growth, structural changes of one-month-old tobacco and pear stems were observed in paraffin sections. *PbXND1* overexpression disrupted the stem meristematic activity, leading to reduced plant height and size of the secondary xylem and vessels, whereas *PbXND1* silencing significantly increased xylem size but reduced vessel size in the pear (Figure S3C–F and Figure 2B–E). To explore the xylem change mechanism, we next quantified the three main components of xylem in the transgenic tobacco and pear. Compared with those of the wild type, the lignin, cellulose and hemicellulose contents were significantly reduced in the PbXND1-overexpression tobacco and pear lines, but they were significantly increased in the PbXND1-RNAi lines (Figure S3G and Figure 2F–H). In addition, microscopic observations showed that *PbXND1* overexpression resulted in the loss of root vessel elements (Figure S3K). Similar results were observed in transgenic pear roots, in which xylem and vessel sizes decreased in the PbXND1-overexpression plants (Figure 2I–L). In brief, our results showed that PbXND1 overexpression resulted in a xylem-deficient extreme dwarf phenotype. 

### 2.3. PbTCP4 Physically Interacts with PbXND1

**Figure 3:**
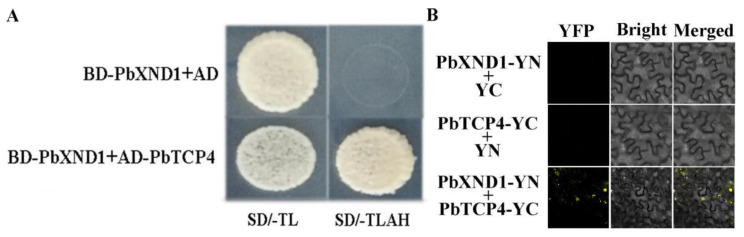
**PbXND1 physically interacts with PbTCP4.** (**A**) Protein interactions between PbXND1 and PbTCP4 were verified by the yeast two-hybrid method. (**B**) Bimolecular fluorescence complementation (BiFC) demonstrated that PbXND1 interacts with PbTCP4 in vivo. Bars, 20 μm.

To understand the action mechanism of PbXND1, we screened for the PbXND1 interaction protein using a pear yeast library. We used the variant PbXND1 N-terminal section as bait for the screening, because the PbXND1 C-terminal section of the protein shows high autoactivation in yeast cells (Figure S4). PbTCP4 was screened and obtained as a candidate PbXND1-interacting protein. Subsequently, we cloned the two genes and carried out Y2H assays to verify the interaction between PbXND1 and PbTCP4. PbTCP4 interacted with PbXND1 in yeast cells (Figure 3A). To further verify the interaction between the PbTCP4 and PbXND1 proteins in plant cells, a bimolecular fluorescence complementation assay was performed on tobacco leaves. The sequences encoding PbTCP4 and PbXND1 were fused to pSPYNE and pSPYCE, respectively. When PbTCP4-NE and PbXND1-CE were transiently co-expressed in tobacco leaves, YFP fluorescence was observed mainly in the nuclei, with a small amount in the cytoplasm (Figure 3B). In summary, our results indicated an interaction between the PbTCP4 and PbXND1 proteins.

### 2.4. PbTCP4 Promotes Xylem Development in Pear Roots

**Figure 4:**
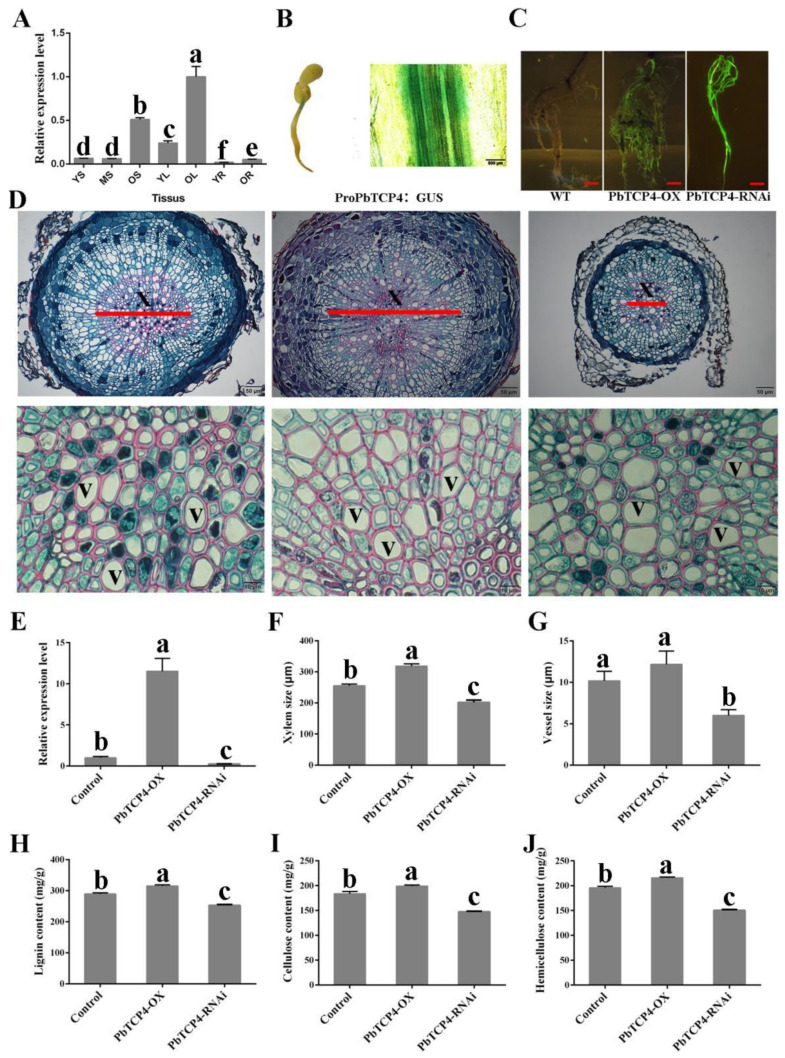
**PbTCP4 promoted xylem development in transgenic pear roots.** (**A**) Expression levels of PbTCP4 in different tissues and developmental stages. YL, young stem; ML, middle stem; OL, old stem; YL, young leaf; OL, old leaf; YR, young root; OR, old root. Data are shown as means ± SD (*n* = 3). (**B**) Activity of the PbTCP4 promoter in pear vascular tissue. (**C**) The growth phenotypes and green fluorescence detection of two-month-old PbTCP4 overexpression and silencing pear roots. Bars, 2 cm. (**D**) Cross sections of roots from pear stained with safranin O and fast green. X, xylem; V, vessel. Bars, 50 µm and 10 µm. (**E**) Relative expression of PbTCP4 in transgenic pear roots. Data are shown as means ±SD (*n* = 3). (**F**) Xylem size of PbTCP4 overexpression and silencing pear roots. (**G**) Vessel size of PbTCP4 overexpression and silencing pear roots. (**H**–**J**) The Lignin, cellulose and hemicellulose contents of transgenic roots. Data are shown as means ± SD (*n* = 3). Different letters denote statistical significance (*p* < 0.05).

We analyzed whether PbTCP4 was involved in xylem development in the pear. We cloned the sequence encoding PbTCP4 from the dwarf hybrid pear seedlings. The resulting phylogenetic tree constructed showed that PbTCP4 was homologous with AtTCP4, and the highly conserved TCP superfamily DNA-binding domain was identified in the PbTCP4 N-terminal sequence (Figure S5). In addition, we analyzed the expression levels of PbTCP4 in pear roots, stems and leaves at different developmental stages. The expression level of PbTCP4 was the highest in the leaves, followed by the stems, and it was the lowest in the roots. The expression level of PbTCP4 increased along with plant tissue maturation and lignification (Figure 4A). In addition, PbTCP4 was mainly expressed in the vascular tissue, as determined by a promoter GUS assay (Figure 4B).

Next, we demonstrated the function of PbTCP4, a protein that interacts with PbXND1. The transgenic roots of the PbTCP4-overexpression and RNAi plants were obtained to validate PbTCP4’s function in the pear. The same methods were used to identify the transgenic roots. Green fluorescence was observed in the transgenic pear roots, whereas green fluorescence was not observed in the non-transgenic roots (Figure 4C). In addition, the PbTCP4 expression level increased in the roots of the overexpression plants and decreased in the roots of the RNAi plants (Figure 4D). To understand whether PbTCP4 participated in the xylem regulatory network, we first performed paraffin sectioning to observe the xylem development (Figure 4E). PbTCP4 overexpression significantly increased the xylem size but did not affect the vessel size in the transgenic pear roots. However, PbTCP4 silencing reduced the xylem and vessel size as compared with the wild-type (Figure 4F,G). Then, the contents of the xylem-related components were determined in the roots. PbTCP4 overexpression significantly increased the contents of lignin, cellulose and hemicellulose in the roots of overexpressing plants, whereas the opposite was found in the roots of silenced plants (Figure 4H–J). Thus, PbTCP4, as a positive regulator, promoted xylem formation in the pear.

### 2.5. PbXND1 Resulted in the Cytoplasm Sequestration of PbTCP4 and Affected Its DNA-Binding Ability

To investigate how *PbXND1* and *PbTCP4* regulate xylem in the pear, we performed a quantitative analysis of the genes associated with xylem development in the transgenic pear, including lignin (*PbCCR*, *PbCCoAOMT*, *PbHCT*, *PbC3H*, *Pb4CL* and *PbCAD*) and cellulose (*PbCESA4*, *PbCESA7* and *PbCESA8*) biosynthetic genes and transcription factors (*PbMYB46*, *PbMYB83*, *PbVND7* and *PbNST1*). PbXND1 overexpression significantly reduced the expression levels of xylem-related genes, except for *PbNST1*. However, PbXND1 silencing promoted the expression of some xylem-related genes (Figure S5A). Additionally, PbTCP4 overexpression promoted the expression of xylem-related genes, and PbTCP4 silencing reduced the expression of xylem-related genes in the transgenic roots (Figure S5B). Among these xylem-related genes, the secondary wall NAC (SWN) genes are master regulators of xylem formation that are located upstream of the MYB transcription factors and xylem-related functional genes. In this study, the expression level of one secondary wall NAC (SWN) gene, *PbVND7*, transformed differently in the transgenic pear in response to PbXND1 and PbTCP4 expression. *PbTCP4* activated the expression of *PbVND7*, whereas *PbXND1* inhibited the expression of *PbVND7* (Figure S5). Thus, we hypothesized that *PbXND1* and *PbTCP4* are involved in *PbVND7*-mediated xylem regulatory networks. 

**Figure 5:**
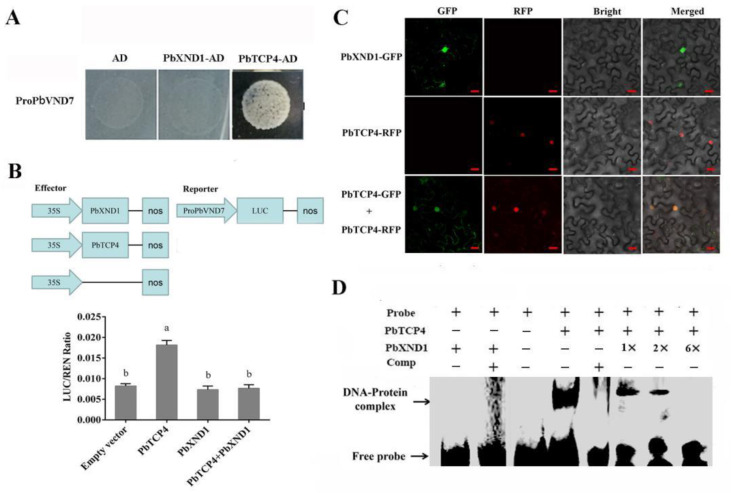
**PbXND1 can repress the transcriptional activity of PbTCP4.** (**A**) Interaction of PbTCP4 with the promoters of PbVND7. (**B**) Vector structure of the effector and reporter used for the tobacco transient expression assays. PbXND1 can repress the transcriptional activity of PbTCP4, as verified by the luciferase assay. Data are shown as means ± SD (*n* = 3). Different letters denote statistical significance (*p* < 0.05). (**C**) Subcellular localization and co-localization of PbTCP4 and PbXND1. Bars, 50 µm. (**D**) PbXND1 affected the DNA-binding ability of PbTCP4 according to EMSA analysis.

To further understand how *PbXND1* and *PbTCP4* are involved in *PbVND7*-mediated xylem regulatory networks, we performed yeast one-hybrid (Y1H) assays. We first analyzed the promoter of *PbVND7*, which contained the GGACCA motif, a reported binding site of TCP4. We used the promoter of *PbVND7* as a bait for analysis, and the Y1H results showed that *PbXND1* could not bind to the promoter. In contrast, PbTCP4 bound to the promoter of ProPbVND7 (Figure 5A). In addition, an effector plasmid, Pro35S:PbTCP4, and reporter plasmid, ProPbVND7-LUC, were constructed, and dual-LUC transient transcriptional activity assays were performed on tobacco leaves (Figure 5B). Transactivation assays showed that PbXND1 did not inhibit the enzyme activities of promoters in ProPbVND7, and PbTCP4 activated the expression of PbVND7 (Figure 5B). These results revealed that *PbVND7* was directly activated by *PbTCP4*.

To understand the PbXND1–PbTCP4 interaction mechanism, Pro35S:PbXND1 and Pro35S:PbTCP4 were co-transformed together with ProPbVND7-LUC. The LUC activity was significantly suppressed, in comparison with co-transformations without PbXND1-62SK. Thus, the transcriptional effect of PbTCP4 on its downstream genes was inhibited by PbXND1 (Figure 5B). These results indicated that PbXND1 acts as a transcriptional repressor of PbTCP4 through protein interactions. To gain further insight into the functions of PbTCP4 and PbXND1 in regulating xylem development, we analyzed the localization of PbTCP4 and PbXND1 in plant cells. The GFP fluorescence signal revealed that PbXND1 was localized to the nuclei and the cytoplasm, and the RFP fluorescence showed that PbTCP4 was localized to the nuclei. Co-localization analyses showed that PbXND1 and PbTCP4 were located in the nuclei and the cytoplasm (Figure 5C). These results indicated that PbXND1 repressed the transcriptional activity of PbTCP4 by affecting the localization of PbTCP4 in the nuclei.

PbXND1 heavily inhibited the PbTCP4 function, but PbXND1 expression resulted only in a small amount of PbTCP4 sequestration in the cytoplasm. Therefore, cytoplasmic sequestration may not be the main cause of PbTCP4 dysfunction. To explore other PbXND1 and PbTCP4 mechanisms, purified PbXND1-HIS and GST-PbTCP4 were used to investigate their DNA-binding activities (Figure S7). PbTCP4, but not PbXND1, directly bound to the PbVND7 promoter. When PbXND1 was incubated with PbTCP4, the PbTCP4 DNA-binding was compromised, as assessed by an electrophoretic mobility shift assay (Figure 5D). Therefore, we concluded that PbXND1 inhibits the function of PbTCP4 mainly by competing for the target DNA-binding site.

**Figure 6:**
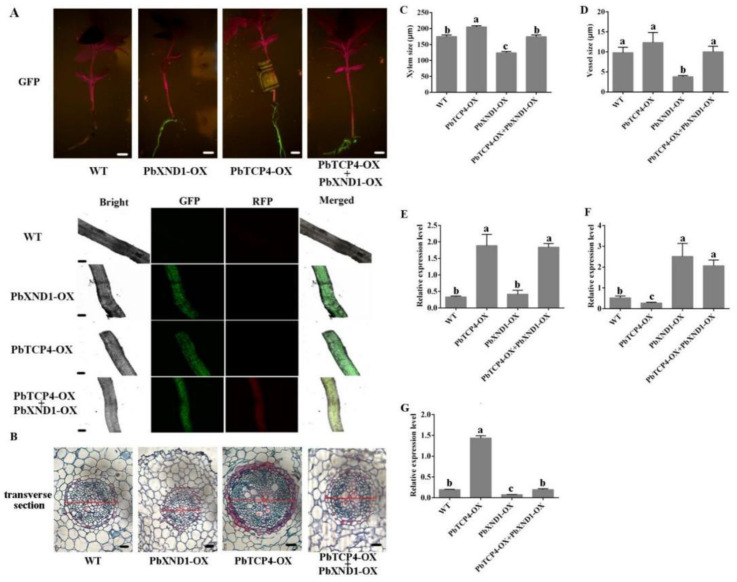
**The xylem development was restored by overexpressing PbTCP4 in the PbXND1-overexpressed pear roots.** (**A**) Fluorescence detection of transgenic pear roots. Bars, 1 cm and 50 µm. (**B**) Transverse section observation of wild-type (WT), PbXND1-OX, PbTCP4-OX and co-expression roots (PbXND1-OX+PbTCP4-OX). X, xylem; V, vessel. Bars, 20 µm. (**C**) Xylem size of pear roots. (**D**) Vessel size of pear roots. Data are shown as means ± SD (*n* = 6). Different letters denote statistical significance (*p* < 0.05). (**E**–**G**) Relative expression of PbTCP4, PbXND1 and PbVND7 in transgenic pear roots. Data are shown as means ± SD (*n* = 3). Different letters denote statistical significance (*p* < 0.05).

To further dissect the relationship between PbXND1 and PbTCP4 in the regulation of xylem, we generated PbXND1 and PbTCP4 co-expression pear roots. Green fluorescence was observed in all the transgenic roots, whereas red fluorescence was only observed in the co-expression roots because PbTCP4 was fused with the RFP protein. The xylem phenotype and vessel size were indistinguishable from those of the wild type (Figure 6B–D). Furthermore, the expression levels of PbXND1 and PbTCP4 increased in the co-expression pear roots as compared with the wild type, whereas that of PbVND7 was similar to that of the wild type (Figure 6E–G). Consequently, the xylem phenotypes of the co-expression pear roots and wild-type roots were similar, indicating an antagonistic effect on the xylem between PbXND1 and PbTCP4. Thus, PbXND1 and PbTCP4 play completely opposite roles in regulating xylem in pear stems and roots.

## 3. Discussion

As demonstrated in a previous study, ‘Red Zaosu’ seedlings exhibited a xylem-deficient dwarf phenotype, and *PbXND1* was identified and obtained from transcriptome data [40]. In this study, the transgenic pears were used to investigate the functional and molecular mechanisms of *PbXND1* and *PbTCP4* involved in regulating xylem development in the pear. In *A. thaliana*, XND1 overexpression disturbed the xylem vessel element formation and resulted in dwarfing [35]. TCP4 promoted xylem formation by activating secondary cell wall biosynthesis [32]. However, whether there is an interaction between PbXND1 and PbTCP4 in regulating xylem development was not reported. 

We isolated PbTCP4 in the pear library as an interacting protein of PbXND1 through Y2H assay, and further confirmed the result through BiFC (Figure 3). A co-expression analysis of *PbTCP4* and *PbXND1* during the pear development and maturation processes showed a strong correlation between the two expressions (Figure S8). The xylem formation increased with the development and tissue maturation of the pear, and PbTCP4 and PbXND1 showed a similar expression pattern in the vascular tissue, as shown by a promoter GUS assay, suggesting a potential unknown interaction between PbTCP4 and PbXND1 in xylem development in the pear (Figure 1F and Figure 4B). 

Subsequently, we verified the function of *PbXND1* and *PbTCP4* in the pear. *PbXND1* overexpression resulted in a xylem-deficient dwarf phenotype, whereas *PbTCP4* overexpression promoted xylem development in the pear (Figure 2 and Figure 4). PbXND1 and PbTCP4 play opposite roles in xylem development, corresponding to PbXND1 and PbTCP4, regulating the expression of xylem-related genes in opposite ways (Figure S6). *TCP4* and *XND1* have been reported to regulate xylem vessel formation by activating or inhibiting *VND7* expression [32,36]. We further revealed that PbTCP4 activated PbVND7 by binding to the promoter of PbVND7, while PbXND1 cannot directly bind to the promoter of PbVND7 (Figure 5A,B). Thus, the inhibition of VND7 promoter activity by XND1 may occur through the indirect inhibition of TCP4. 

Synergistic or antagonistic interactions between proteins play important roles in plant growth and development. *XND1* is expressed in a pattern that is similar to that of *NST1* and interacts with *NST1* to inhibit the transcriptional activation activity of *NST1* [41]. The XND1 protein interacts with the VND family of genes and affects their regulation of downstream secondary wall-related genes. Furthermore, XND1 affects the entry of VND6 into the nucleus, resulting in VND6 being unable to activate the expression of downstream genes [39]. In this study, we demonstrated the existence of interactions between the PbXND1 and PbTCP4 proteins. Dual-LUC assays showed that PbXND1 affected the activation of PbTCP4 (Figure 5B). To further investigate the action mechanism of the PbXND1–PbTCP4 complex, we determined the subcellular localization and co-localization of PbXND1 and PbTCP4 in tobacco leaves. Unlike the exclusive nuclear localization of PbTCP4, PbXND1 was localized to the nuclei and the cytoplasm. Similarly, PbXND1 co-expression with PbTCP4 resulted in a small amount of PbTCP4 sequestration in the cytoplasm, but it did not affect the localization of the PbXND1 protein (Figure 5C). Thus, PbXND1 may interact with PbTCP4 and prevent it from entering the nucleus in order to perform transcriptional activation. The interaction between TCP4 and AtWRI1 represses the transcriptional activity of AtWRI1 and reduces the synthesis of fatty acids in Arabidopsis [42]. Therefore, we hypothesized that PbTCP4 promoted xylem formation in the pear by inhibiting the transcriptional activity of PbXND1. Here, we showed that the PbXND1 expression-related cytoplasmic sequestration of PbTCP4 may be a minor factor. Furthermore, we concluded that PbXND1 inhibits the function of PbTCP4 mainly by competing for the target DNA-binding site, as assessed using electrophoretic mobility shift assays (Figure 5D). Current studies suggest that XND1 inhibits the transcriptional activation of NST1, but other studies suggest that XND1 inhibits the VNDs family genes mainly through cytoplasmic isolation [39,41]. However, these studies mainly focused on the XND1 and NAC transcription factor families, including VNDs and NST1, which are related to secondary wall development. In this study, we verified the interaction between PbXND1 and PbTCP4 proteins and identified a new regulatory mechanism in the xylem development of the pear. Overall, XND1-regulated xylem development is complex and involves multiple key regulators of xylem development.

**Figure 7:**
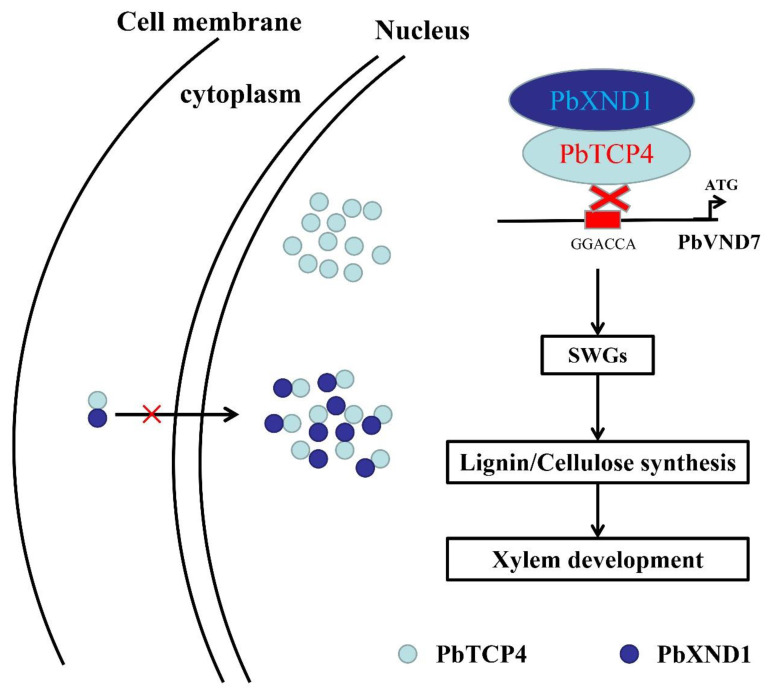
**A model of PbXND1 and PbTCP4 in the xylem regulatory pathways.** Unlike the exclusive nuclear localization of PbTCP4, most of the PbXND1 protein is localized to the nucleus, and only a small fraction is localized to the cytoplasm. Therefore, PbXND1 co-expression with PbTCP4 resulted in a small amount of PbTCP4 sequestration in the cytoplasm and thereby prevented it from activating gene expression. Moreover, the PbXND1-PbTCP4 protein complex affects the DNA-binding ability of PbTCP4 to the PbVND7 promoter. Overall, PbXND1 affects the function of PbTCP4 mainly by affecting the DNA-binding ability of PbTCP4, and a small amount of cytoplasm sequestration of PbTCP4 may be a minor factor in the pear. Finally, the overexpression of PbXND1 reduced the expression of secondary wall-related genes and the synthesis of xylem-related components, thereby inhibiting xylem development in pears.

Previous studies have shown that XND1 overexpression results in extreme dwarfism, associated with the absence of xylem development [35]. Other studies have further demonstrated that XND1 inhibits the function of multiple key regulators of xylem development through protein interactions, including VND family genes and NST1 [39,41]. In this study, we discovered a previously unreported mechanism of action between PbXND1 and PbTCP4, and this finding opens the door to understanding the complex regulatory network of xylem development centered on PbXND1. The pathways and specific molecular mechanisms by which PbXND1 and PbTCP4 regulate xylem formation are summarized in Figure 7. In contrast to the reported mechanism of action of XND1, we report a novel mechanism of action of PbXND1 in this study. PbXND1 affects the function of PbTCP4 mainly by affecting the DNA-binding ability of PbTCP4, and the cytoplasmic sequestration of PbTCP4 is only a minor factor. Plant NAC transcription factors are also actively involved in the regulation of plant hormones. For example, the Arabidopsis NAC transcription factor JUB1 reduces plant height by regulating GA/BR metabolism and signal transduction [43]. OsNAC2 encodes an NAC transcription factor that affects plant height through mediating the gibberellic acid pathway in rice [44]. Therefore, PbXND1 may also be involved in the hormonal pathways in plants. In conclusion, our study provides new theoretical support for the extreme dwarfism associated with the absence of xylem caused by PbXND1, and it has significant reference value for the breeding of dwarf varieties and dwarf rootstocks of the pear.

## 4. Methods

### 4.1. Plant Materials

*Pyrus betulifolia* Bunge cv Duli seedlings were used for the tissue culture derived from mature seed germination. *Pyrus betulaefolia* Bunge seeds were cultured in a medium (MS medium supplemented with 0.4 mg/L 6-BA, 0.1 mg/L IBA) in dark conditions at 25 °C.

The seeds of the wild-type (NC89) and transgenic tobacco were sown into soil. After germination, the tobacco seedlings were individually transplanted into pots, and the growing conditions of the greenhouse were controlled at 23 ± 2 °C with a 16/8 h light/dark cycle.

### 4.2. RNA Extraction and qRT-PCR

Samples from different tissues and developmental stages of *Pyrus betulaefolia* Bunge and the transgenic pear and tobacco were used to analyze the tissue-specific expression and the differential expression of downstream genes. RNA extraction and qRT-PCR were the same as the findings described by Wang et al. [45]. Primers are listed in Table S1.

### 4.3. Vectors and Transformation

PbXND1 was cloned into the PK7-203 and PK7WWG2D vector to generate overexpression and silence construct PK7-203-35S-PbXND1 and PK7WWG2D-RNAi- PbXND1, respectively. The overexpression and RNAi vectors of PbXND1 were then introduced into *Agrobacterium tumefaciens* strain EHA105 for tobacco and *Pyrus betulaefolia* transformation according to the methods of Zheng et al. and Xiao et al. [2,46]. The PbTCP4 overexpression vector was transferred into *Agrobacterium rhizogenes* K599, the methods of root transformation according to Xiao et al. [46]. All amplification primers were listed in Table S1. 

### 4.4. Histological Analysis

The stems of Pyrus betulaefolia and tobacco were cut at 0.5 cm above the ground, and roots with diameters of 0.5–1 mm were selected for the xylem analyses. Three stem and three root segments per plant were fixed with FAA for 24 h. Then, paraffin sections of the stems and roots were performed by embedding, sectioning and dyeing. The specific steps were consistent with those described by Wang et al. [45]. A fluorescence microscope (BX63, Olympus, Tokyo, Japan) was used for the anatomical observations, and the xylem and vessel cells were measured using cellSens software.

### 4.5. Xylem Components

Fresh stem segments of 2-month-old *Pyrus betulaefolia* and tobacco plants were cut and five segments of each line were used for the determination of the xylem components. The total lignin, cellulose and hemicellulose were extracted using the lignin content kit (Comin, Suzhou, China), cellulose content kit (Comin, Suzhou, China) and hemicellulose content kit (Comin, Suzhou, China), respectively, and the three components were measured using an ultraviolet spectrophotometer. 

### 4.6. Dual-Luciferase Assay

The 2000 bp promoters of PbVND7 were inserted into the plasmid pGreenII 0800-LUC and used as the reporter vector. The full-length CDS sequences of PbXND1 and PbTCP4 were cloned and inserted into the plasmid pGreenII 0029-62SK as the effector. The dual-luciferase assay was performed in accordance with the method of Wang et al. [45]. All amplification primers are listed in Table S1.

### 4.7. Interaction Analysis

The interaction between PbXND1 and PbTCP4 was verified using the yeast two-hybrid method. The DNA-binding domain of PbXND1 was fused to the GAL4 DNA-binding domain in the pGBKT7 vector as the bait, and the full-length PbTCP4 was inserted into the pGADT7 as the prey. The bait and prey were co-transformed into the yeast strain Y2H Gold and the protein–protein interaction was tested using the medium (-Trp/-Leu/-His/-Ade).

BiFC experiments were performed to further verify the protein interactions. The CDS sequences of PbXND1 and PbTCP4 were inserted into the pSPYCE-35S and pSPYNE-35S, respectively. The fused plasmids were transformed into GV3101, and the transformed strains were co-injected into the leaves of *Nicotiana benthamiana*. Fluorescence was observed through a laser scanning confocal microscope (TCS-SP8 SR, Leica, Wetzlar, Germany). All amplification primers are listed in Table S1.

### 4.8. Subcellular Localization and Co-Localization

The CDS sequences of PbXND1 and PbTCP4 were inserted into the 2300-GFP and 1300-RFP, respectively. The transformed strains were injected into the leaves of *Nicotiana benthamiana*. The fluorescence was observed through a laser scanning confocal microscope (TCS-SP8 SR, Leica, Wetzlar, Germany).

### 4.9. EMSA

An EMSA assay was used for the analysis of the effect of PbXND1 on the PbTCP4 DNA-binding activity. PbXND1-HIS and PbTCP4-GST fusion proteins were obtained using GST-Resin and Ni-NTA His Bind Resin, respectively (7sea biotech, Shanghai, China). Recombinant PbXND1-HIS and PbTCP4-GST proteins were detected using SDS-PAGE. EMSA was performed to detect the binding of the recombinant PbXND1-HIS and PbTCP4-GST proteins with the PbVND7 promoters in vitro, through which labeled PbVND7 promoter fragments containing the GGACCA motif were produced using biotin-labeled oligonucleotides (General Biol, Anhui, China) or unlabeled oligonucleotides (as unlabeled competitors). The primers are listed in Table S1. The EMSA reactions were performed as described by Zheng et al. [47]. Each experiment was independently repeated three times.

### 4.10. Statistical Analysis

SPSS software (IBM, Armonk, NY, USA) was used to perform the analysis of variance by Fisher’s LSD or Student’s *t*-test, and value thresholds of *p* < 0.05 were suggested to be statistically significant.

### 4.11. Accession Numbers

The gene sequences can be downloaded from the NCBI database, and the genes and their accession numbers are indicated in Table S2.

## Data Availability

The author responsible for the distribution of the materials integral to the findings presented in this article, in accordance with the policy described in the Instructions for Authors, is Lingfei Xu (lingfxu2013@sina.com).

## Supplementary Materials

The following supporting information can be downloaded at: https://www.mdpi.com/article/10.3390/ijms23158699/s1.
