# Supplementary material for: PbXND1 Results in a Xylem-Deficient Dwarf Phenotype through Interaction with PbTCP4 in Pear (Pyrus bretschneideri Rehd.)
Source: Int J Mol Sci. 2022 Aug 4;23(15):8699. doi: 10.3390/ijms23158699 (PMC9369282; doi:10.3390/ijms23158699)
Supplement: Supplementary file 1 [file ijms-23-08699-s001.zip › ijms-1818457-supplementary.pdf]

Table S1 Primers used in this study

Abbreviation	Forward (5' to 3')	Reverse (5' to 3')	Function
PbXND1	GGGCAAGCAGTGGTATTTCTA	CACAATCTGAGGCTGACGAAG	RT - PCR
MYC	GAACAGAAACTGATCTCTGAAG		
PbTCP4	GTGGAGACCCACAATCCGA	GTGGAGACCCACAATCCGA	
Pb4CL	CTTGGACAGGGGTATGGAATG	AGCGAAGCACCCGTATCAG	
PbC3H	TGATTACTGCGGGTATGGACA	AGCATTAGTGGTGTTGGAGGG	
PbHCT	GACGCCAAAGAAGGCACTG	GGGTAGAACGGCACAAGAGC	
PbCCR	CAGAACAAATGGTGGAGCCC	CCGATTGACGACGTGAAAAC	
PbCCoAOMT	TACCCAAGGGAGCCTGAATC	AGTAGCCAGTGTAGACCCCAATC	
PbCAD	CCTCCTTGTTGGTTGCTGC	CCGCCTTGGGTGGTTTT	
PbVND7	TACAAGGGACGGGCACCTAA	CCGGCATACAACCCATCCTT	
PbMYB46	CGGAGGGTTCATTAGTCCCG	CAAACATGTGGTTGAGGATCG	
PbMYB83	AGTCCCACATGCTTCCCA	CTCAATGCTTCTGCTTTCG	
PbCES4	ACGTTGTCCATGCCACTGAA	AGTCATAACAAGGCCGGCAA	
PbCES7	CAACGCCGCTGCCAGAT	TGAGCGTGCGATGAGTATGTG	
PbCES8	AACACCCCAACTTCCACACC	CCACTTGCCAATCTGGGACT	
PbNST1	GATATGCAGCAACTGCAGGC	AGATTCGGCAAACCACCCAT	
PbXND1	tagaactagtggatccATGGGAGATAACCAGTTTAAGCTC	cggtatcgataagcttTTAATTTGGCAAACTTATTTCATC	LUC assay
PbTCP4	tagaactagtggatccATGGGGGATTCTCACCACC	cggtatcgataagcttTCAATGGCGAGAATTGGAGGAAG	
PbVND7	cggtatcgataagcttATTCCTTCTTCAGTATCTCTCCA	tagaactagtggatccGCACCTGCATATCAGGCA	
PbXND1	agctcggtacccggggatccATGGGAGATAACCAGTTTAAGCTC	CttgctcaccatggtgtcgacATTTGGCAAACTTATTTCATC	Localizati-on
PbTCP4	ccggggatcctctagaATGGGGGATTCTCACCACC	aggaggccatgtcgacATGGCGAGAATTGGAGGAAG	
PbXND1	atggccatggaggccgaattcATGGGAGATAACCAGTTTAAGCTC	ccgctgcaggtcgacggatccTTATAGACGATACTCATGCA	Yeast validation
PbXND1-C	atggccatggaggccgaattcACAGAATACAGTGGATATG		
PbXND1-N		gccgctgcaggtcgacTTATTTGGGTTGCCCTC	
PbTCP4	gaggccagtgaattcATGGGGGATTCTCACCACC	cgagctcgatggatccATGGCGAGAATTGGAGGAAG	
PbVND7	aaaagcttgaattcgagctcATTCCTTCTTCAGTATCTCTCCA	gagcacatgcctcgagGCACCTGCATATCAGGCA	
PbXND1	ctggcgcgccactagtggatccATGGGAGATAACCAGTTTAAGCTC	tcccgggagcggtaccctcgagTTATTTGGGTTGCCCTC	BIFC Assay
PbTCP4	ctggcgcgccactagtggatccATGGGGGATTCTCACCACC	tcccgggagcggtaccctcgagATGGCGAGAATTGGAGGAAG	
PbXND1-OX	ggggacaagtttgtacaaaaaagcaggctgcATG	ggggaccactttgtacaagaaagctgggtctgtTT	Plant

	GGAGATAACCAGTTTAAGCTC	AATTTGGCAAACTTATTTCATC	**transform-ation vector**
PbXND1-RNAi	ggggacaagtttgtacaaaaaagcaggctgcTATGATCCGTGGGAACTAAA	ggggaccactttgtacaagaaagctgggtctgtGGAAGAAGAAGCACAATCTG	
PbTCP4-OX	ggggacaagtttgtacaaaaaagcaggctgcATGGGGGATTCTCACCACC	ggggaccactttgtacaagaaagctgggtcTCAATGGCGAGAATTGGAGGAAG	
PbTCP4-RNAi	ggggacaagtttgtacaaaaaagcaggctgcAACAGCGGAGACGGAGATAGC	ggggaccactttgtacaagaaagctgggtctgtATGATGGTCGTGAGAAATGG	
PbXND1-HIS	tatcggatccgaattcATGGGAGATAACCAGTTTAAGCTC	gtgcggccgcaagcttATTTGGCAAACTTATTTCATC	**prokaryotic expression**
GST-PbTCP4	gggggcccccctgggatccATGGGGGATTCTCACCACC	gatgcggccgctcgagTCAATGGCGAGAATTGGAGGAAG	
PbVND7-Bio-3’	ACATGCAGGACCACTCACAGACATGCAGGACCACTCACAGACATGCAGGACCACTCACAGACATGCAGGACCACTCACAG	CTGTGAGTGGTCCTGCATGTCTGTGAGTGGTCCTGCATGTCTGTGAGTGGTCCTGCATGTCTGTGAGTGGTCCTGCATGT	EMSA
PbVND7	ACATGCAGGACCACTCACAGACATGCAGGACCACTCACAGACATGCAGGACCACTCACAGACATGCAGGACCACTCACAG	CTGTGAGTGGTCCTGCATGTCTGTGAGTGGTCCTGCATGTCTGTGAGTGGTCCTGCATGTCTGTGAGTGGTCCTGCATGT	

Note: Lowercase show vector adaptor

Table S2 Accession numbers

The genes sequences can downloaded from the NCBI database, and the genes and their accession numbers are as follows:

**Abbreviation**	Accession number	**Abbreviation**	Accession number
PbXND1	LOC103926530	AtNST1	At2g46770
PbTCP4	LOC103932452	AtNST2	At3g61910
PbVND7	LOC103964117	AtSND3	At1g28470
PbMYB46	LOC103963897	AtSND2	At4g28500
PbMYB83	LOC103954864	AtATAF2	At5g08790
PbNST1	LOC103956366	AtATAF1	At1g01720
PbCESA4	LOC103959701	AtXND1	At5g64530
PbCESA7	LOC103956004	AtTCP10	At2g31070
PbCESA8	LOC103943043	AtTCP13	At3g02150
PbC3H	LOC103944996	AtTCP14	AT3G47620

PbHCT	LOC103945380	AtTCP15	AT1G69690
PbCCR	LOC103945355	AtTCP3	At1g53230
Pb4CL	LOC103927375	AtTCP4	At3g15030
PbCCoAOMT	LOC103933993	AtTCP21	At5g08330
PbCAD	LOC103928508	AtTCP23	At1g35560
AtVND6	At5g62380	AtTCP9	At2g45680
AtVND7	At1g71930	AtTCP18	At3g18550
AtVND3	At5g66300	AtTCP5	At5g60970
AtVND1	At2g18060	AtTCP2	At4g18390
AtVND5	At1g62700	AtTCP19	At5g51910
AtVND2	At4g36160	AtTCP16	At3g45150
AtVND4	At1g12260	AtTCP20	At3g27010
AtSND1	At1g32770	AtTCP24	At1g30210
AtTCP7	At5g23280	AtTCP12	At1g68800
AtTCP1	At1g67260	AtTCP6	At5g41030
AtTCP11	At2g37000	AtTCP22	At1g72010
AtTCP8	At1g58100	AtTCP17	At5g08070

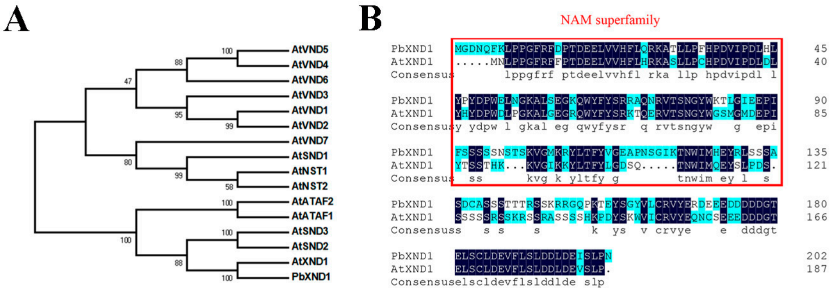

Figure S1 Phylogenetic analysis and sequence alignment of PbXND1. A-B Phylogenetic tree and sequence alignment of PbXND1. The neighbor-joining tree and a bootstrap test with 1000 iterations were constructed using the MEGA7. The NAC DNA-binding functional regions are marked in the red box.

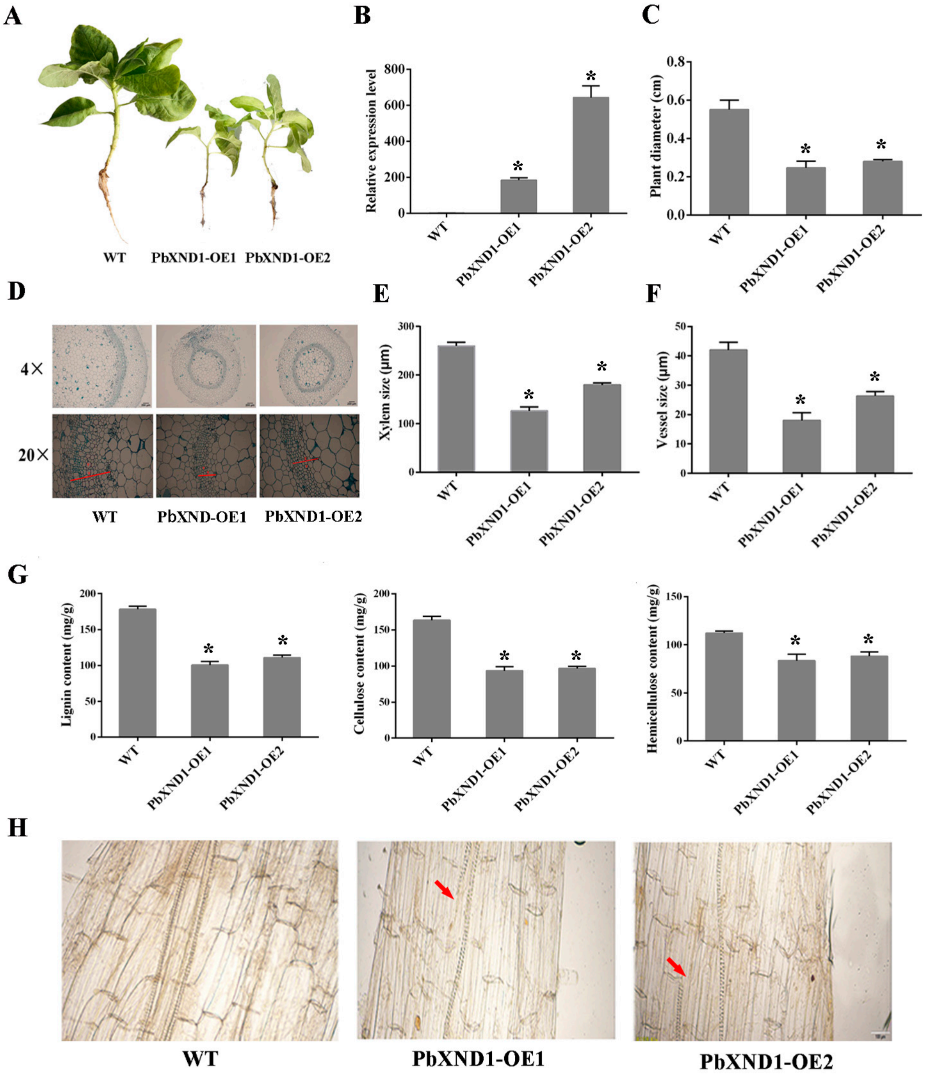

Figure S2 PbXND1 inhibited xylem development in transgenic tobacco. A The growth phenotypes of one-month-old PbXND1 overexpressed tobacco plants and wild-type plants. Bars=1 cm. **B** Relative expression of PbXND1. **C** plant diameter in tobacco plants. Data are means ± sd (n=3). **D** Cross sections of stems from tobacco plants stained with safranin O and fast green. X, xylem; V, vessel. Bars, 200 μm and 50 μm. **E** Xylem size. **F** vessel size. **G** Lignin, Cellulose and Hemicellulose content. One week-old tobacco stems were used as experimental material, Data are means ± sd (n=6). One-way ANOVA (Tukey test) was performed, and statistically significant differences are indicated by *$P < 0.05$. **H** Microscopic observation of tobacco root system. The red arrow indicates the vessel element.

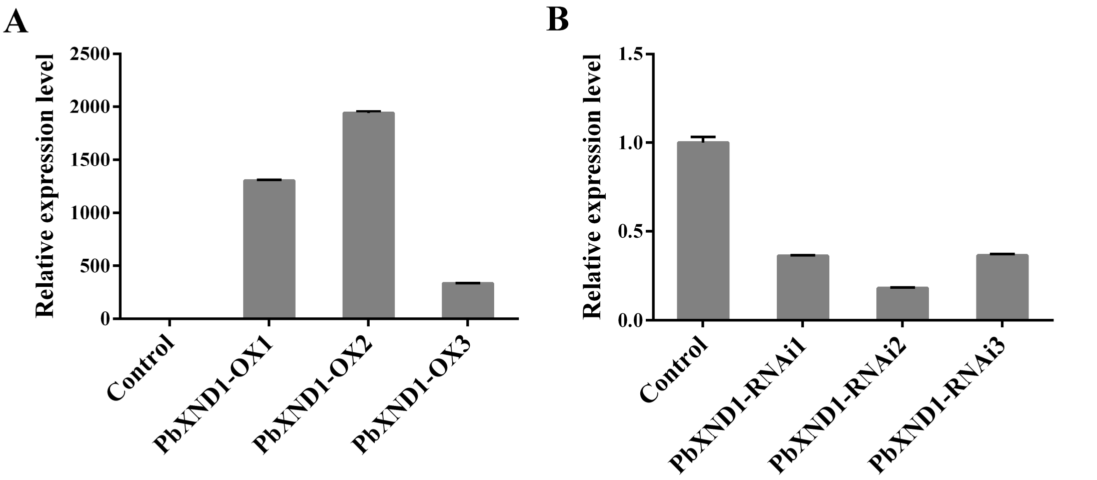

Figure S3 (A,B) The expression level of PbXND1 in the overexpression and RNAi pear. Data are means ± sd (n=3).

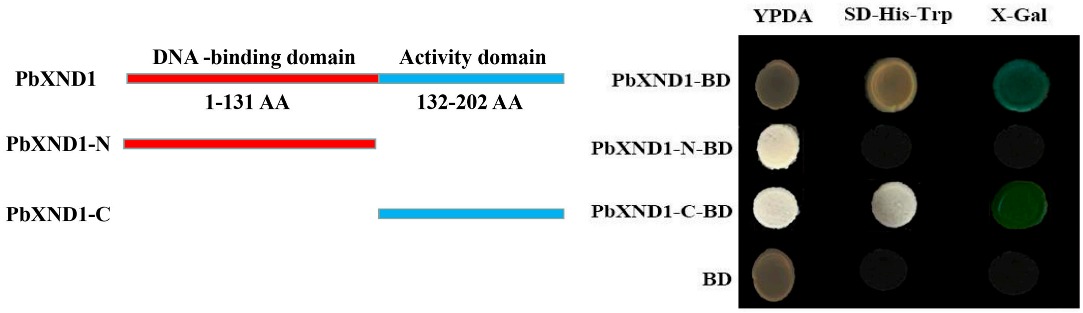

Figure S4 Transcriptional activation assays of PbXND1.

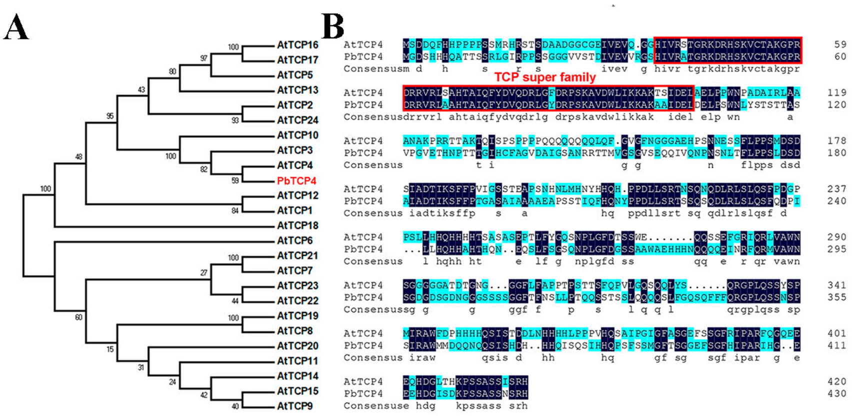

Figure S5 Phylogenetic analysis and sequence alignment of PbTCP4. A-B Phylogenetic tree and sequence alignment of and PbTCP4. The neighbor-joining tree and a bootstrap test with 1000 iterations were constructed using the MEGA7. The TCP DNA-binding functional regions are marked in the red box.

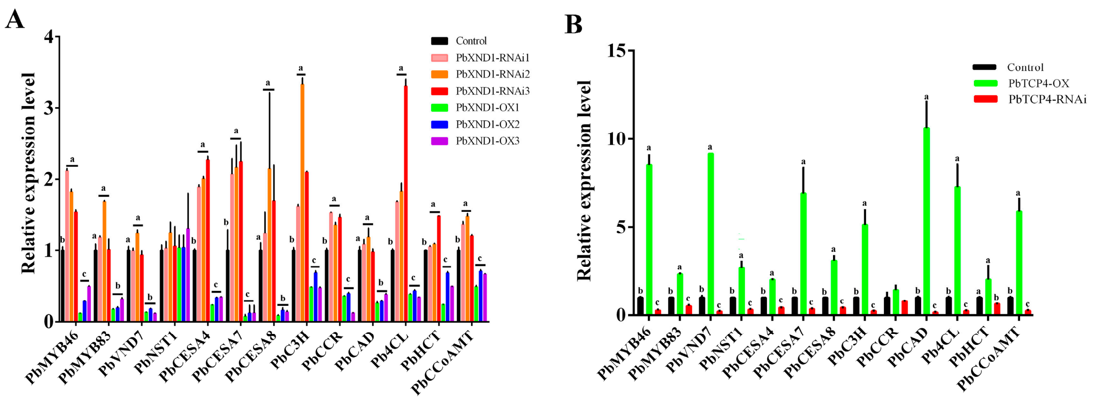

Figure S6 PbXND1 and PbTCP4 regulate the expression of xylem-related genes in pear. A PbXND1 inhibited the expression of secondary wall related transcription factors and functional genes. **B** PbTCP4 promoted the expression of secondary wall related transcription factors and functional genes. Data are means ± SD (n=3). Different letters denote statistical significance ($p < 0.05$).

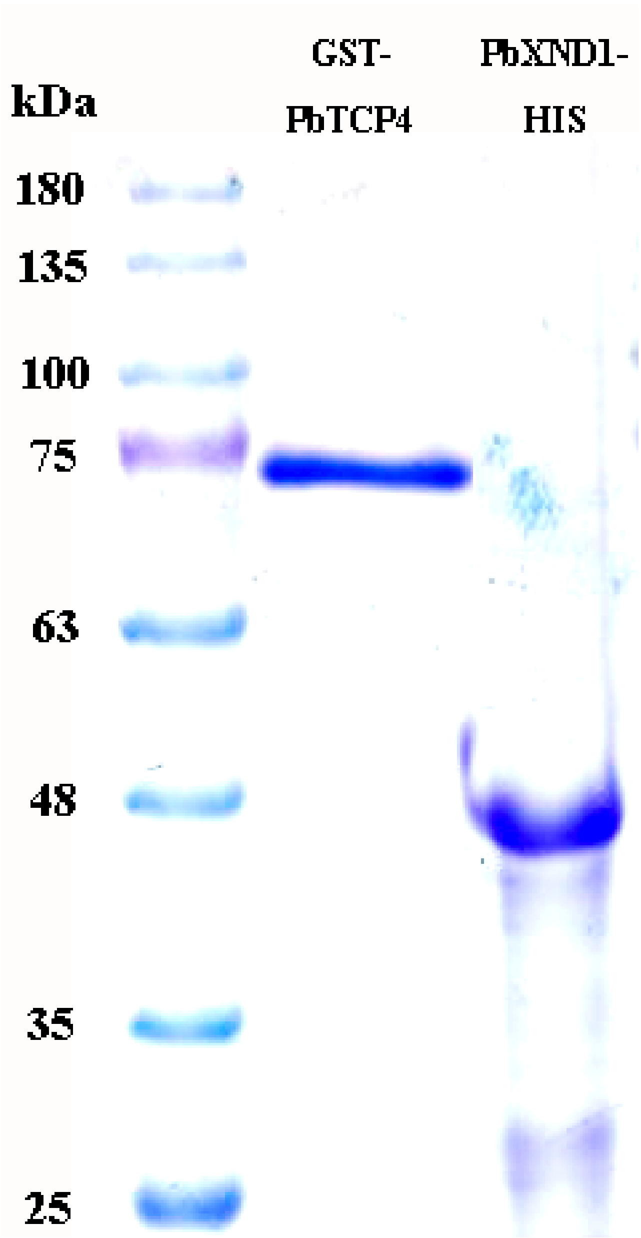

Figure S7 The purified GST-PbTCP4 and PbXND1-HIS was detected by SDS-PAGE.

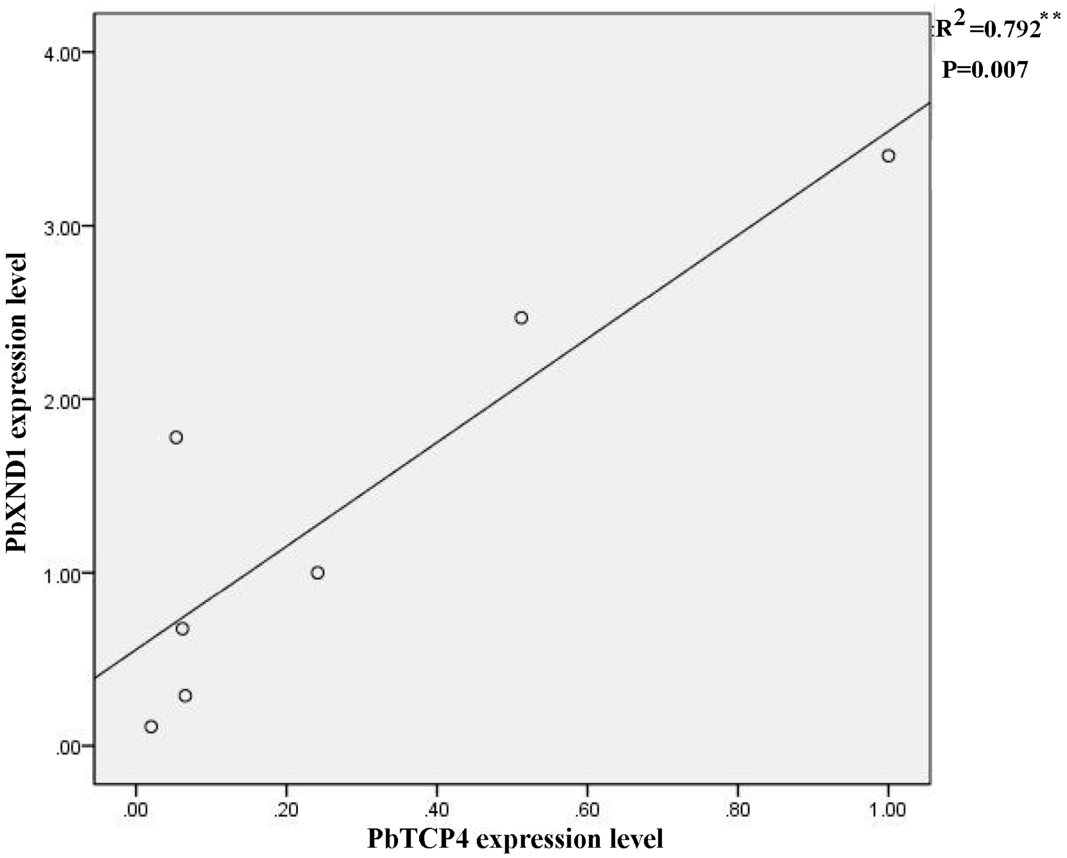

Figure S8 Correlation analysis of PbXND1 and PbTCP4 in different tissues and development stages. ** denote statistical significance ($p < 0.01$).
